# Importance of occupation for SARS-CoV-2 seroprevalence and COVID-19 vaccination among correctional workers in Quebec, Canada: A cross-sectional study

**DOI:** 10.3389/fpubh.2022.1021871

**Published:** 2022-11-09

**Authors:** Nadine Kronfli, Camille Dussault, Mathieu Maheu-Giroux, Alexandros Halavrezos, Sylvie Chalifoux, Hyejin Park, Lina Del Balso, Matthew P. Cheng, Joseph Cox

**Affiliations:** ^1^Centre for Outcomes Research and Evaluation, Research Institute of the McGill University Health Centre, Montreal, QC, Canada; ^2^Department of Medicine, Division of Infectious Diseases and Chronic Viral Illness Service, McGill University Health Centre, Montreal, QC, Canada; ^3^Department of Epidemiology and Biostatistics, School of Population and Global Health, Faculty of Medicine and Health Sciences, McGill University, Montreal, QC, Canada; ^4^Department of Medicine, Divisions of Infectious Diseases and Medical Microbiology, McGill University Health Centre, Montreal, QC, Canada

**Keywords:** SARS-CoV-2, seroprevalence, antibody, correctional workers, prison, vaccine

## Abstract

**Background:**

Correctional workers are at increased risk of SARS-CoV-2 infection. We examined the seroprevalence of SARS-CoV-2, determined the effects of carceral and occupational exposures on seropositivity, and explored predictors of COVID-19 vaccine uptake among correctional workers in Quebec, Canada.

**Methods:**

We conducted a cross-sectional seroprevalence study in three provincial prisons. The primary and secondary outcomes were SARS-CoV-2 antibody seropositivity (Roche Elecsys^®^ serology test) and self-reported COVID-19 vaccination status (“fully vaccinated” defined as two doses or prior infection plus one dose), respectively. Poisson regression models with robust standard error were used to examine the effect of occupational variables with SARS-CoV-2 seropositivity and predictors of COVID-19 vaccine uptake. Estimates are presented as crude and adjusted prevalence ratios (aPR) with 95% confidence intervals (95% CI).

**Results:**

From 14 July to 15 November 2021, 105/600 (18%) correctional workers tested positive across three prisons (range 11–21%); 76% were fully vaccinated. Seropositivity was affected by prison occupation (aPR 1.59, 95% CI 1.11–2.27 for correctional officers vs. all other occupations) and low perceived concern of SARS-CoV-2 acquisition (aPR 1.62, 95% CI 1.11–2.38 for not/hardly worried vs. somewhat/extremely worried). Predictors of being fully vaccinated included race/ethnicity (aPR 0.86, 95% CI 0.76–0.99 for visible minority vs. White), presence of comorbidities (aPR 1.14, 95% CI 1.02–1.28 for > 2 vs. none), and prison occupation (aPR 0.82, 95% CI 0.73–0.92 for correctional officers vs. all other occupations).

**Conclusions:**

Correctional officers were most likely to have acquired SARS-CoV-2, but least likely to be vaccinated, underscoring the importance of addressing both occupational risks and COVID-19 vaccine hesitancy to mitigate future outbreaks.

## Introduction

Canadian correctional facilities have witnessed many large outbreaks of the novel severe acute respiratory syndrome coronavirus (SARS-CoV-2) since the beginning of the pandemic, affecting both incarcerated individuals and correctional workers alike (1, 2). Several studies have demonstrated that COVID-19 case rates among incarcerated individuals and correctional workers constantly exceeded those of surrounding communities during the pandemic (3–6), underscoring the prison environment as particularly conducive to SARS-CoV-2 transmission. Correctional workers have experienced many of the same infrastructural catalysts to SARS-CoV-2 transmission as individuals in prison, including inadequate ventilation, unhygienic conditions, and difficulties in accessing and implementing effective infection prevention and control measures (7–11) – occupational risks that are further heightened by daily community exposures. In fact, studies investigating the temporal relationship between COVID-19 and the broader carceral community have emphasized the vital role of correctional workers in infection control (12).

There is a paucity of research devoted to correctional workers during the COVID-19 pandemic. For example, very few SARS-CoV-2 seroprevalence studies have been conducted among correctional workers (13, 14). The few that have, were either conducted in middle-income countries (13), where restricted mitigation strategies likely contributed to higher SARS-CoV-2 seroprevalence, or failed to differentiate seroprevalence by correctional occupation (14), the latter of which can drive public health recommendations. While studies have used SARS-CoV-2 PCR testing to attempt to capture the disease burden among correctional workers (4–6, 15–18), they likely underestimated the true extent of SARS-CoV-2 exposure because of decreased symptom disclosure due to mandatory quarantining, a finite number of remunerated sick days, and a focus on symptom-based testing. These reasons further underscore the need for large seroprevalence studies.

By July 2021, Quebec reported over 370,000 confirmed COVID-19 infections and 11,000 COVID-19 related deaths among the general population (19). Cognizant of the heightened risk of SARS-CoV-2 transmission in correctional settings, “resident and staff of congregate settings” were prioritized for early COVID-19 vaccination in December 2020 by the Canadian National Advisory Committee on Immunization (20). COVID-19 vaccine coverage among correctional workers remains lower than surrounding communities in many countries (21–23); however, very few studies have sought to understand individual- and occupation-related characteristics associated with vaccine uptake in these settings (21). To address these important knowledge gaps, we sought to examine the SARS-CoV-2 seroprevalence among correctional workers in Quebec provincial prisons, determine the effects of carceral and occupational exposures on SARS-CoV-2 seropositivity, and explore predictors of COVID-19 vaccine uptake.

## Methods

### Study design and setting

We conducted an observational cross-sectional study in three provincial prisons, representing 42% of correctional workers in Quebec (24). The three selected study sites were: *l'Établissement de détention de Montréal* (EDM), *l'Établissement de détention de Rivière-des-Prairies* (EDRDP), and l'É*tablissement de détention de Saint-Jérôme* (EDSJ). EDM and EDRDP are both located in Montreal, the Quebec epicenter of the SARS-CoV-2 pandemic, whereas EDSJ is located in the Laurentian region. Characteristics of each prison are presented in Supplementary Table 1.

### Participants

Correctional workers over the age of 18 and who were able to consent in either English or French were invited to participate in this study. Those who were in isolation with COVID-19 or quarantined at home as a contact of a positive case were excluded. No financial compensation was provided as study participation occurred during working hours. The McGill University Health Centre Research Ethics Board (#2022–7801) and the Direction régionale des services correctionnels du Québec (#2020–11240) approved this study.

Study recruitment occurred between 14 July to 15 November 2021. The number of participants recruited from each site was proportional to the site's total number of correctional workers. The minimum sample size was 578, based on a SARS-CoV-2 seroprevalence similar to that of blood donors after the second wave in Quebec [i.e., 10.5% (25)] with a 2.5% absolute margin of error (26).

### Data collection

All employees at each of the three study sites were invited to participate, and those meeting the eligibility criteria were included. Recruitment strategies included study promotion *via* posters and email communication to all employees (~1,400) by each prison director describing the study. Efforts to encourage underrepresented workers were taken (*via* direct engagement of the research team) to ensure diversity in participation across prison occupations (i.e., administration, kitchen, janitorial, and managerial). Prospective participants were instructed to meet the research team in the designated office, where they provided written consent for participation, and were given 24 h to complete a self-administered paper questionnaire. This included questions on sociodemographic characteristics, working environment, COVID-19 risk factors and exposures, and COVID-19 vaccination status.

The Roche Elecsys^®^ serology test was used to detect SARS-CoV-2 antibodies. This highly specific (>99.8%) and sensitive (99.5%) serology test that targets SARS-CoV-2 nucleocapsid proteins (27) was deliberately chosen as it does not cross-react with vaccine-induced antibodies. Whole blood samples were collected, centrifuged on-site within 2 h, and sent to Sacré-Coeur Hospital (Montreal) for analysis. Test results were sent to all participants by email within 72 h of serology testing by the research team.

### Outcomes of interest

The primary outcome measure was SARS-CoV-2 seropositivity, measured as a positive result to the Roche Elecsys^®^ serology test. The secondary outcome measure was COVID-19 vaccination status, measured using a self-reported history to both a prior positive SARS-CoV-2 PCR test and the total number of COVID-19 vaccines received. Correctional workers were offered vaccination in Quebec on 30 April 2021, but many were eligible as of 1 March 2021, when age-based population-wide COVID-19 vaccination began (28). At the time of the study, the Quebec Ministry of Health and Social Services considered individuals to be “fully vaccinated” if those who previously tested positive for SARS-CoV-2 received at least one dose of a COVID-19 vaccine approved by Health Canada (Pfizer BioNTech, Moderna, AstraZeneca, Janssen, or Covisheild) (29) and those who never tested positive for SARS-CoV-2 received two doses (unless Janssen) (28). We dichotomized COVID-19 vaccination status into “fully vaccinated” and “not fully vaccinated” using this definition.

### Independent variables of interest

Independent variables were selected based on a literature review of factors associated with SARS-CoV-2 seroprevalence and vaccine uptake among employees of congregate settings, including correctional facilities (6, 14, 16, 21, 30). Variables were grouped into the following categories: sociodemographic (age, sex, ethnicity, education level, and yearly income), clinical (self-reported history of prior SARS-CoV-2 PCR testing, COVID-19 symptoms, medical comorbidities, and COVID-19 vaccination), and carceral characteristics (provincial prison, prison occupation, number of workdays, meal consumption with others at work, direct daily contact with people in prison, ability to physically distance, and perceived concern of SARS-CoV-2 acquisition in prison). Prison occupation was dichotomized into two occupational groups: correctional officers (COs) vs. all others based on COs' greater direct contact with incarcerated people (30–32). Number of workdays, meal consumption, direct daily contact, ability to physically distance, and perceived concern of SARS-CoV-2 acquisition in prison were measured by participant responses to the following questions, respectively: “*In a typical month, how many days per month do you work in this prison?*” (< 21 days vs. 21-27 days vs. ≥ 28 days), “*Since March 2020, who have you primarily had meals with when at work*” (alone vs. with others), “*In a typical shift, how often are you in direct contact (i.e.*,<* 2 meters for at least 10 min, with or without a mask) with incarcerated people?*” (< 10% vs. 10–49% vs. ≥ 50%), “*Currently, does the prison environment allow for physical distancing with other prison staff and inmates (i.e., enough space to keep a safe distance from other people, plexiglass barriers, etc.)?*” (all the time vs. almost all the time vs. some of the time vs. rarely), and “*I am worried that people around me will infect me with the virus*” (extremely/somewhat worried vs. neutral vs. hardly/not worried).

### Statistical analyses

Summary statistics were calculated to describe the study sample and graphs were generated to depict patterns of SARS-CoV-2 seroprevalence and COVID-19 vaccination status by prison occupation.

For the primary outcome, Poisson regression models with robust standard errors, clustered by prison sites, were used to examine the effects of four carceral exposures of interest (prison occupation, meal consumption with others at work, direct daily contact with people in prison, and perceived concern of SARS-CoV-2 acquisition in prison) on SARS-CoV-2 seropositivity. Directed acyclic graphs (33) were used to depict relationships between these exposures and the outcomes and identify confounders for inclusion in multivariable regression models (see Supplementary Figure 1). Since the effect of an exposure on the outcome can be mediated, separate multivariable models were constructed for each carceral and occupational exposure of interest and their total effect on the estimated outcome, resulting in four sets of adjustment variables. For the secondary outcome, Poisson regression models with robust standard errors, clustered by prison sites, were used to explore potential predictors of COVID-19 vaccine status among correctional workers. In this case, we were interested in predictors of vaccine uptake to identify characteristics associated with uptake and model selection was performed using goodness of fit statistics. The final multivariable model was chosen using the quasi-likelihood under the independence model criterion (QIC) (34, 35). Both outcomes were reported as crude and adjusted prevalence ratios (aPR) with 95% confidence intervals (95% CI).

Multiple imputation was performed for all models to reduce bias attributable to missing observations, resulting in five imputed data sets and pooled estimates using Rubin's rule. All analyses were performed using R statistical software (version 4.1.2) and the “geepack,” “MuMIn,” and “mice” packages.

## Results

### Sample characteristics

A total of 600 correctional workers were enrolled in this study (n = 310 at EDM, n = 163 at EDRDP, and *n* = 127 at EDSJ), representing a participation rate of 57% (600/1,050). Overall, the median age was 43 years, 45% were male, 75% self-identified as White, and 76% had a college level education or higher (Table 1). A minority (13%) reported a history of COVID-19, 48% reported at least one comorbidity, and 12% were never vaccinated against COVID-19. More than half (53%) of correctional workers were COs and over one-third (38%) reported no concern of SARS-CoV-2 acquisition in prison (37% among COs).

**Table 1:** Participants' socio-demographic and behavioral characteristics stratified by SARS-CoV-2 antibody screening test.

	**Negative** **(*n* = 495)**	**Positive** **(*n* = 105)**	**Total** **(*n* = 600)**
**Sociodemographic characteristics**			
Age, years			
Median (Q1–Q3)	43 (33–52)	44 (33–49)	43 (33–51)
Age category – *n* (%)			
18–29	72 (14%)	14 (13%)	86 (14%)
30–39	111 (22%)	31 (30%)	142 (24%)
40–49	156 (32%)	33 (31%)	189 (31%)
50 and over	154 (31%)	26 (25%)	180 (30%)
*Missing*	*2 (1%)*	*1 (1%)*	*3 (1%)*
Sex – *n* (%)			
Male	217 (44%)	54 (51%)	271 (45%)
Female	276 (55%)	50 (48%)	326 (54%)
*Missing*	*2 (1%)*	*1 (1%)*	*3 (1%)*
Race/ethnicity – *n* (%)			
White	379 (76%)	72 (69%)	451 (75%)
Visible minority^a^	108 (22%)	29 (27%)	137 (23%)
*Missing*	*8 (2%)*	*4 (4%)*	*12 (2%)*
Education level – *n* (%)			
High school or trade certificate	117 (23%)	15 (14%)	132 (22%)
College diploma	206 (42%)	50 (48%)	256 (43%)
University or higher	164 (33%)	36 (34%)	200 (33%)
*Missing*	*8 (2%)*	*4 (4%)*	*12 (2%)*
Personal gross yearly income^b^ (CAD) – *n* (%)		
Less than $60,000	128 (26%)	16 (15%)	144 (24%)
$60,000–$89,999	242 (49%)	55 (52%)	297 (50%)
$90,000 or more	96 (19%)	27 (26%)	123 (20%)
*Missing*	*29 (6%)*	*7 (7%)*	*36 (6%)*
**Clinical characteristics**			
History of SARS-CoV-2 PCR testing – *n* (%)			
No previous PCR test	69 (14%)	6 (6%)	75 (12%)
Negative PCR test	419 (84%)	22 (21%)	441 (74%)
Positive PCR test	5 (1%)	76 (72%)	81 (13%)
*Missing*	*2 (1%)*	*1 (1%)*	*3 (1%)*
History of COVID-19 symptoms^c^ – *n* (%)		
No	180 (36%)	14 (13%)	194 (32%)
Yes	311 (63%)	89 (85%)	400 (67%)
*Missing*	*4 (1%)*	*2 (2%)*	*6 (1%)*
Medical comorbidities^d^ – *n* (%)			
None	245 (50%)	49 (46%)	294 (49%)
1	150 (30%)	29 (28%)	179 (30%)
≥ 2	86 (17%)	25 (24%)	111 (18%)
*Missing*	*14 (3%)*	*2 (2%)*	*16 (3%)*
COVID-19 vaccination – *n* (%)			
Not vaccinated	50 (10%)	24 (23%)	74 (12%)
1 dose	56 (11%)	26 (25%)	82 (14%)
2 doses	381 (77%)	49 (47%)	430 (72%)
*Missing*	*8 (2%)*	*6 (5%)*	*14 (2%)*
COVID-19 vaccine status – *n* (%)			
Not fully vaccinated	104 (21%)	26 (25%)	130 (22%)
Fully vaccinated	383 (77%)	73 (70%)	456 (76%)
*Missing*	*8 (2%)*	*6 (6%)*	*14 (2%)*
**Carceral characteristics**			
Prison – *n* (%)			
EDRDP	137 (28%)	26 (25%)	163 (27%)
EDM	245 (49%)	65 (62%)	310 (52%)
EDSJ	113 (23%)	14 (13%)	127 (21%)
Prison occupation – *n* (%)			
Administration	52 (10%)	7 (7%)	59 (10%)
Correctional officer	247 (50%)	70 (67%)	317 (53%)
Healthcare provider	31 (6%)	8 (7%)	39 (6%)
Kitchen/dining	28 (6%)	4 (4%)	32 (5%)
Manager	68 (14%)	12 (11%)	80 (13%)
Other^e^	66 (13%)	4 (4%)	70 (12%)
*Missing*	*3 (1%)*	*0 (0%)*	*3 (1%)*
Number of workdays in prison per month – *n* (%)	
< 21	209 (42%)	47 (45%)	256 (42%)
21–27	229 (46%)	40 (38%)	269 (45%)
≥ 28	49 (10%)	15 (14%)	64 (11%)
*Missing*	*8 (2%)*	*3 (3%)*	*11 (2%)*
Meal consumption with others in prison – *n* (%)		
Alone	167 (34%)	29 (27%)	196 (33%)
With others^**f**^	321 (65%)	71 (68%)	398 (65%)
*Missing*	*7 (1%)*	*5 (5%)*	*12 (2%)*
Direct daily contact with people in prison^g^ – *n* (%)		
< 10%	270 (55%)	52 (49%)	322 (54%)
10–49%	125 (25%)	21 (20%)	146 (24%)
>50%	100 (20%)	32 (31%)	132 (22%)
Ability to physically distance in prison^h^ – *n* (%)		
All the time	94 (19%)	13 (12%)	107 (18%)
Almost all the time	189 (38%)	33 (31%)	222 (37%)
Some of the time	97 (20%)	25 (24%)	122 (20%)
Rarely	105 (21%)	33 (31%)	138 (23%)
*Missing*	*10 (2%)*	*1 (1%)*	*11 (2%)*
Perceived concern of SARS-CoV-2 acquisition from others in prison – *n* (%)		
Somewhat or extremely worried	206 (42%)	36 (34%)	242 (40%)
Neutral	110 (22%)	17 (16%)	127 (21%)
Not or hardly worried	179 (36%)	51 (49%)	230 (38%)
*Missing*	*0 (0%)*	*1 (1%)*	*1 (0.2%)*

EDM, Établissement de détention de Montréal; EDRDP, Établissement de détention de Rivière-des-Prairies; EDSJ, Établissement de détention de Saint-Jérôme.

^a^Includes Black, Latin American, Arab, Asian, Indigenous, and Asian.

^b^Refers to total annual income (CAD) from all paid work and all other sources before taxes and other deductions in the year prior to incarceration.

^c^Includes fever, chills, headache, sore throat, new or worsening cough, stuffy nose/congestion, difficulty breathing/shortness of breath, loss of smell or taste, fatigue, weakness, confusion, diarrhea, muscle pain, vomiting and nausea.

^d^Includes hypertension, diabetes, obesity (based on body mass index), asthma, chronic lung disease, chronic heart disease, chronic kidney disease, liver disease, cancer, chronic blood disorder, chronic neurological disorder, immunocompromised (HIV), immunocompromised (other).

^e^Includes probation agents, community workers, janitorial staff, building maintenance, teachers, pastoral, laundry, library, researchers, information technology, and students.

^f^Defined as the presence/absence of meal sharing with other correctional employees from the same or other sectors while working in prison.

^g^Defined as being within < 2 meters for at least 10 min, with or without a mask.

^h^Defined as whether the prison environment allows for physical distancing with other prison staff and incarcerated individuals (i.e., enough space to keep safe distance from other people, plexi-glass barriers).

### SARS-CoV-2 seropositivity

**Figure 1:**
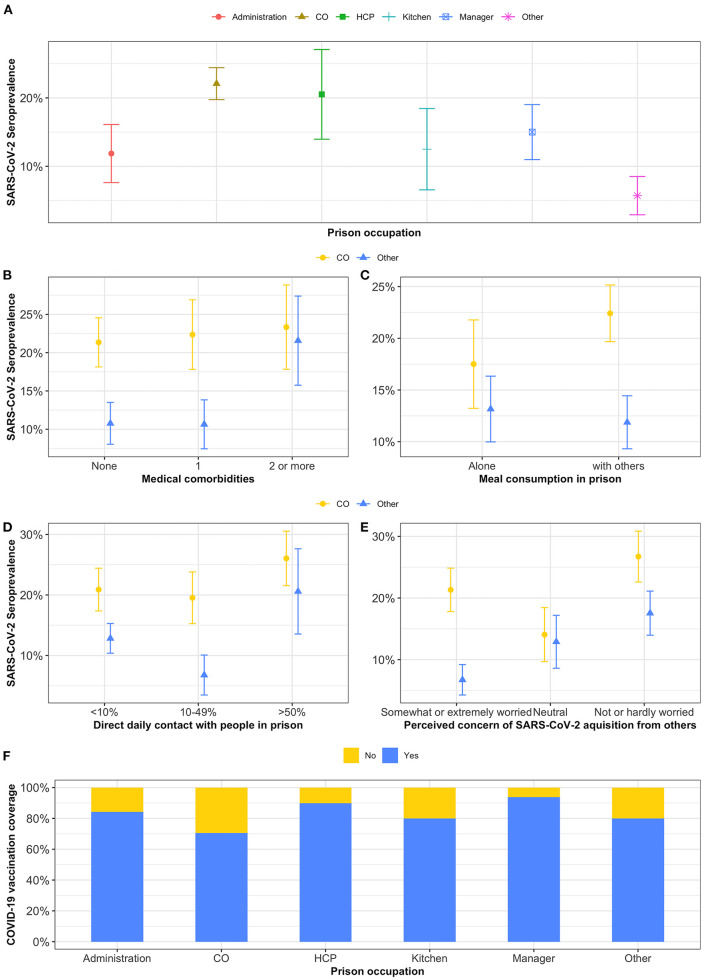
**(A-F)** Point prevalence of SARS-CoV-2 and COVID-19 vaccination coverage by prison occupation. CO, correctional officers; HCP, healthcare providers; Other, other occupations (i.e., probation agents, community workers, janitorial staff, building maintenance, teachers, pastoral, laundry, library, researchers, information technology, and students).

A total of 105 (18%) participants were seropositive: 65 (21%) at EDM, 26 (16%) at EDRDP, and 14 (11%) at EDSJ (Table 1). Of these, 98 (93%) reported having at least one previous SARS-CoV-2 PCR test, with 76 (72%) testing positive and 22 (21%) testing negative. Among those who were seropositive, 14 (13%) reported no prior COVID-19 symptoms. Among those who were seronegative, 424/495 (86%) underwent prior SARS-CoV-2 PCR testing with 1% reporting a previous positive result. SARS-CoV-2 seroprevalence varied by prison occupation (Figure 1A) and was consistently higher among COs irrespective of the variable of interest (Figure 1). Overall, 70/317 (22%) of COs were seropositive vs. 35/280 (13%) for all other occupations.

Risk factors for SARS-CoV-2 seropositivity identified in univariate and multivariable analyses are presented in Table 2. In multivariable analyses, seropositivity was higher among COs (aPR 1.59, 95% CI 1.11–2.27 vs. all other occupations) and those with low perceived concern of SARS-CoV-2 acquisition in prison (aPR 1.62, 95% CI 1.11–2.38 vs. somewhat or extremely worried). Neither meal consumption with others at work nor direct daily contact with people in prison were significantly associated with SARS-CoV-2 seroprevalence.

**Table 2:** Unadjusted and adjusted associations between carceral exposures of interest and SARS-CoV-2 seropositivity among correctional workers in three provincial prisons in Quebec, Canada (2021).

**Model**	**Variables**	**Prevalence ratio**	**95% confidence interval**	**Adjusted prevalence ratio**	**95% confidence interval**
**1** ^**a**^	**Prison occupation**				
	Other^b^	Reference	Reference	Reference	Reference
	Correctional officer	1.77	1.24–2.52	1.59	1.11–2.27
**2** ^**c**^	**Meal consumption in prison** ^**d**^				
	Alone	Reference	Reference	Reference	Reference
	With others	1.21	0.80–1.82	1.16	0.78–1.72
**3** ^**e**^	**Direct daily contact with people in prison** ^**f**^				
	< 10%	Reference	Reference	Reference	Reference
	10–49%	0.89	0.57–1.39	0.84	0.54–1.29
	≥ 50%	1.50	1.02–2.22	1.29	0.86–1.93
**4** ^**g**^	**Perceived concern of SARS-CoV-2 acquisition from others in prison**				
	Somewhat or extremely worried	Reference	Reference	Reference	Reference
	Neutral	0.89	0.52–1.53	0.91	0.53–1.56
	Not or hardly worried	1.49	1.03–2.17	1.62	1.11–2.38

^a^Adjusted for age, sex, race/ethnicity, and education level.

^b^Includes administration, healthcare provider, kitchen, manager and other occupations (i.e., probation agents, community workers, janitorial staff, building maintenance, teachers, pastoral, laundry, library, researchers, information technology, and students).

^c^Adjusted for sex, race/ethnicity, prison occupation, and perceived concern of SARS-CoV-2 acquisition from others.

^d^Defined as the presence/absence of meal sharing with other correctional employees from the same or other sectors while working in prison.

^e^Adjusted for medical comorbidities, number of workdays in prison per month, and prison occupation.

^f^Defined as being within < 2 meters for at least 10 min, with or without a mask.

^g^Adjusted for age, sex, race/ethnicity, education level, medical comorbidities, prison occupation and direct daily contact with people in prison.

### COVID-19 vaccination status

Three-quarters (76%) of all correctional workers were fully vaccinated against COVID-19 (i.e., two doses or prior infection plus one dose). All but one received at least one dose of an mRNA vaccine (Pfizer-BioNTech or Moderna). Among those who reported a history of a positive SARS-CoV-2 test, 61/81 (75%) received at least one dose of a COVID-19 vaccine, and among those who reported no history of a positive SARS-CoV-2 test, 394/516 (76%) received two doses of a COVID-19 vaccine. The occupations with the highest proportion of fully vaccinated workers were managers (94%), healthcare providers (90%), administration (82%), and kitchen staff (80%) (see Supplementary Figure 1f, Supplementary Table 2). A total of 130 (22%) correctional workers were not fully vaccinated, 91 (70%) of whom were COs. This differed by prison: 45, 11, and 2% of COs were not fully vaccinated at EDM, ERDP, and ESJ, respectively.

Correlates associated with COVID-19 vaccination identified in univariate and multivariable analyses are presented in Table 3. In multivariable analyses, COs (aPR 0.82, 95% CI 0.73–0.92; vs. all other occupations) and those who self-identified as a visible minority (aPR 0.86, 95% CI 0.74–0.99; vs. White) were less likely to be fully vaccinated against COVID-19. Conversely, individuals with two or more medical comorbidities (aPR 1.14, 95% CI 1.02–1.28; vs. none) were more likely to be fully vaccinated against COVID-19. Age, sex, and education were not predictors of COVID-19 vaccine uptake among correctional workers.

**Table 3:** Univariate and multivariable analyses of predictors of COVID-19 vaccination status among correctional workers in three provincial prisons in Quebec, Canada (2021).

**Variables**	**Prevalence ratio**	**95% confidence interval**	**Adjusted prevalence ratio**	**95% confidence interval**
Age category				
18–29	Reference	Reference	Reference	Reference
30–39	1.12	0.94–1.32	1.15	0.98–1.35
40–49	1.16	0.97–1.39	1.16	0.98–1.37
≥ 50	1.12	0.95–1.31	1.14	0.98–1.33
Sex				
Male	Reference	Reference	Reference	Reference
Female	1.09	0.99–1.21	1.08	0.98–1.20
Race/ethnicity				
White	Reference	Reference	Reference	Reference
Visible minority^a^	0.83	0.73–0.96	0.86	0.76–0.99
Education level				
High school or trade certificate	Reference	Reference	Reference	Reference
College diploma	0.96	0.86–1.07	1.05	0.94–1.18
University or higher	0.88	0.76–1.00	0.96	0.83–1.10
Medical comorbidities^b^				
None	Reference	Reference	Reference	Reference
1	1.10	1.00–1.20	1.08	0.98–1.19
≥ 2	1.15	1.03–1.28	1.14	1.02–1.28
Prison occupation				
Other^c^	Reference	Reference	Reference	Reference
Correctional officer	0.82	0.74–0.90	0.82	0.73–0.92

^a^Includes Black, Latin American, Arab, Asian, Indigenous, and Asian.

^b^Includes hypertension, diabetes, obesity (based on body mass index), asthma, chronic lung disease, chronic heart disease, chronic kidney disease, liver disease, cancer, chronic blood disorder, chronic neurological disorder, immunocompromised (HIV), immunocompromised (Other).

^c^Includes administration, healthcare provider, kitchen, manager and other occupations (i.e., probation agents, community workers, janitorial staff, building maintenance, teachers, pastoral, laundry, library, researchers, information technology, and students).

## Discussion

We present the first description of SARS-CoV-2 seroprevalence and of predictors of COVID-19 vaccine uptake among Canadian correctional workers. We observed a seroprevalence (18%) that was approximately 2-fold higher than in blood donors after the second SARS-CoV-2 wave in Quebec (i.e., 10.5%), underscoring the high-risk posed by both the carceral environment and occupational exposures. We also found that COs were approximately 1.6 times more likely to have been previously exposed to SARS-CoV-2, although they were 1.2 times less likely to be fully vaccinated compared to other correctional workers. Finally, we identified that low perceived concern of SARS-CoV-2 acquisition from others in prison was associated with a higher seroprevalence. Our results have important implications on public health policies focused on mitigating future COVID-19 outbreaks in correctional settings.

While we observed a relatively high SARS-CoV-2 seroprevalence among correctional workers, it ranged between 11 and 21% depending on the prison. This variability may be explained by several reasons including the size of preceding prison-based and regional SARS-CoV-2 outbreaks and the timing of study recruitment. For example, there were 134, 63, and 27 correctional workers and 266, 113, and 34 incarcerated individuals who were PCR positive at EDM, EDSJ, and EDRDP, respectively, during the first three waves of the COVID-19 pandemic in Quebec (25 February 2020 – 13 July 2021). Furthermore, there was a very large outbreak at EDRDP during our recruitment period, which likely contributed to the higher seroprevalence at EDRDP (compared to EDSJ) despite having the fewest cases in the first three waves. Finally, each prison has a variable number of correctional workers and incarcerated individuals, and is different in size and spatial organization, further impacting differences in seroprevalence. This variability was also observed during our preceding seroprevalence study among incarcerated individuals at the same three sites (27% at EDM, 19% at EDSJ, and 15% at EDRDP) (3).

Very few studies exploring the risk of infectious diseases in correctional settings have focused on correctional workers despite having shared health and safety hazards as those incarcerated (36). Although a limited number of studies have shown that COVID-19 case rates among correctional workers were closer to those among the incarcerated population than the general population (3–6), confirming the high-risk prison environment, very few have investigated the seroprevalence of COVID-19 by correctional occupation. Toblin et al. failed to find an association between occupation and SARS-CoV-2 PCR positivity (16), while Duque et al. demonstrated that COs were almost twice as likely to be SARS-CoV-2 antibody positive compared to healthcare providers (13). The former study likely failed to find an association as they relied on PCR rather than seroprevalence testing, which unlikely captured the true disease burden, while the latter study failed to include a broad spectrum of correctional occupations. We found that COs were 1.6 times more likely to have been previously exposed to SARS-CoV-2 compared to all other employees, a finding that highlights that COs are a high-risk occupational group who may require targeted interventions around COVID-19 risk perception and mitigation strategies (37), as has been suggested for people in prison (38, 39), and as a population for priority vaccination in countries where this is not the case.

Prison occupation was also predictive of lower vaccine uptake, as has been found in another study (21), with COs being 1.2 times less likely to be vaccinated compared to other employees. In fact, COs comprised 70% of all workers who were not fully vaccinated. Although many countries have prioritized correctional workers for COVID-19 vaccination (40), many correctional facilities have witnessed higher proportions of vaccinated inmates than employees (21, 23). Furthermore, mandatory vaccination of correctional workers has been suggested as a COVID-19 mitigation strategy but has been met with legislative pushback in the United States and, in Canada, has been unsuccessful at the provincial/territorial levels. Correctional Services Canada, which oversees 43 federal prisons across the country, will require that workers attest to their vaccination status by 29 October 2022 (to date, only 77% of workers have completed their attestation) (41). Those who fail to attest will be placed on administrative leave without pay (41), and those who have attested may be subject to random verification. Instead, additional studies are needed to understand reasons for vaccine hesitancy among correctional workers, with the goal of addressing those that may be modifiable (42). Finally, COVID-19 vaccination status differed considerably by prison; 45, 11, and 2% of COs were not fully vaccinated at EDM, ERDP, and ESJ, respectively. Additional studies could seek to explore reasons for such discrepancies and apply best practices nationally and internationally.

There are study limitations to highlight. First, our study sites were chosen based on practical considerations, restricting the study population to correctional workers in three of 16 provincial prisons in Quebec. Moreover, different-sized outbreaks occurred at all three sites prior to and during study recruitment, reducing generalizability to other facilities without SARS-CoV-2 outbreaks, or outside of Quebec. While our sampling method may have introduced selection bias, direct engagement strategies were employed by our research team to recruit a diverse representation of correctional workers. In fact, the distribution of our study participants' occupations was comparable to the overall distribution of correctional roles across the three sites (see Supplementary Table 1). Second, the reported seroprevalence of SARS-CoV-2 was likely underestimated for several reasons. We only included correctional workers present at the three prison facilities during the recruitment period, thus excluding those in isolation due to suspected or confirmed COVID-19 infection, on vacation or on sick leave. Furthermore, as recruitment occurred longitudinally (over 4 months) and not simultaneously across study sites, seroreversion may have occurred in our study population as antibodies induced by SARS-CoV-2 infection waned over time. Third, the nature of the study design (cross-sectional) prevents our ability to determine where and when participants acquired SARS-CoV-2 infection, be it in prison or the community. While our questionnaire inquired where previous testing occurred and about test results, various unmeasured variables related to SARS-CoV-2 infection and vaccination were not collected, thus influencing our inferences. Furthermore, the measured variables were self-reported by study participants, which may have introduced information bias. Fourth, COVID-19 vaccination was not included in the DAGs or in the regression analyses of the primary outcome to avoid reverse causation, resulting in uninterpretable total effects on the estimated outcome. The first COVID-19 vaccine dose occurred after the last reported positive SARS-CoV-2 PCR test for the majority (75/81; 93%) of participants who reported a positive test. That said, DAGs were used to identify confounders and obtain total effects, missing data were addressed with multiple imputation.

In conclusion, we found a high prevalence of exposure to SARS-CoV-2 among correctional workers in the provincial prison system in Quebec. COs were the most likely to have acquired SARS-CoV-2, but the least likely to be fully vaccinated, underscoring the importance of addressing both occupational risks, COVID-19 risk misconceptions, and vaccine hesitancy to mitigate future COVID-19 outbreaks.

## Data availability statement

The datasets presented in this article are not readily available because of ethical and privacy restrictions. Requests to access the datasets should be directed to NK, nadine.kronfli@mcgill.ca.

## Ethics statement

This study was reviewed and approved by McGill University Health Centre Research Ethics Board (#2022–7801) and the Direction régionale des services correctionnels du Québec (#2020–12493). The patients/participants provided their written informed consent to participate in this study.

## Author contributions

NK, CD, and JC contributed to the conception of the work. NK, CD, and MM-G contributed to the study design and performed data analyses. AH, SC, HP, and LB facilitated data acquisition. NK, CD, MM-G, AH, SC, HP, LB, MC, and JC interpreted the data. NK and CD drafted the manuscript. All of the authors revised it critically for important intellectual content, gave final approval of the version to be published, and agreed to be accountable for all aspects of the work.

## Funding

This work was supported by the *Public Health Agency of Canada* through the Sero-Surveillance and Research (COVID-19 Immunity Task Force Initiative) Program (#2021-HQ-000103). The funders had no role in the study design, data collection, data analysis, data interpretation, or writing of the report. NK is supported by a career award from the Fonds de Recherche Québec –* Santé (FRQ-S; Junior 1*). MM-G is supported by a Canada Research Chair (Tier 2) in *Population Health Modeling*.

## Conflict of interest

Author NK reports research funding from Gilead Sciences, advisory fees from Gilead Sciences, ViiV Healthcare, Merck and Abbvie, and speaker fees from Gilead Sciences, Abbvie and Merck, all outside of the submitted work. Author MM-G reports an investigator-sponsored research grant from Gilead Sciences Inc. Author MM-G reports contractual arrangements with the World Health Organization, the Institut national de santé publique du Québec, and the Institut 19 d'excellence en santé et services sociaux du Québec, all outside of the submitted work. Author MC reports grants from the McGill Interdisciplinary Initiative in Infection and Immunity and from the Canadian Institutes of Health Research. Author MC reports personal fees from GEn1E Lifesciences and form nplex biosciences, both outside the submitted work. Author MC is the co-founder of Kanvas Biosciences, Inc. and owns equity in the company. Author MC has a pending patent for Methods for detecting tissue damage, graft versus host disease, and infections using cell-free DNA profiling pending, and a pending patent for Methods for assessing the severity and progression of SARS-CoV-2 infections using cell-free DNA. Author JC has research funding from ViiV Healthcare and Gilead Sciences, and reports remuneration for advisory work (ViiV Healthcare, Gilead Sciences and Merck Canada), outside the submitted work. The remaining authors declare that the research was conducted in the absence of any commercial or financial relationships that could be construed as a potential conflict of interest.

## Publisher's note

All claims expressed in this article are solely those of the authors and do not necessarily represent those of their affiliated organizations, or those of the publisher, the editors and the reviewers. Any product that may be evaluated in this article, or claim that may be made by its manufacturer, is not guaranteed or endorsed by the publisher.
