# Supplementary material for: Importance of occupation for SARS-CoV-2 seroprevalence and COVID-19 vaccination among correctional workers in Quebec, Canada: A cross-sectional study
Source: Front Public Health. 2022 Nov 9;10:1021871. doi: 10.3389/fpubh.2022.1021871 (PMC9683106; doi:10.3389/fpubh.2022.1021871)
Supplement: Supplementary file 1 [file Data_Sheet_1.docx]

**Supplementary Material**

Supplementary Table **S1.** Characteristics of the three study sites (Quebec, Canada).

Supplementary Table **S2.** Participants’ sociodemographic and behavioural characteristics stratified by prison occupation.

Supplementary Figure **S1.** Direct acyclic graphs of the relationships between the carceral exposure of interest on SARS-CoV-2 seropositivity.

**Supplementary Table S1. Characteristics of the three prison sites in Quebec, Canada.**

|  | **Établissement de détention de Montréal** | **Établissement de détention de Rivière-des-Prairies** | **Établissement de détention de Saint-Jérome** |
| --- | --- | --- | --- |
| **Location** | Montreal | Montreal | Laurentian region |
| **Prison capacity (1)** | 1,400 | 541 | 587 |
| **Number of SARS-CoV-2 cases before recruitment period (i.e., March 2020 to July 13, 2021) (2)** | 134 employees  266 incarcerated individuals | 27 employees  34 incarcerated individuals | 63 employees  113 incarcerated individuals |
| **Number of SARS-CoV-2 cases during recruitment period (i.e., July 14-November 15, 2021)** | 5 employees  1 incarcerated individual | 15 employees  68 incarcerated individuals | 2 employees  8 incarcerated individuals |
| **Recruitment period** | July 14-August 6, 2021 | October 8-14 and November 1-15, 2021 | September 17- October 6, 2021 |
| **Number of full- and part-time employees pre-COVID** | 724 | 526 | 321 |
| Administration | 48 (7%) | 35 (7%) | 19 (6%) |
| Correctional officer | 554 (77%) | 402 (76%) | 236 (74%) |
| Manager | 65 (9%) | 52 (10%) | 29 (9%) |
| Professional^a^ | 27 (4%) | 11 (2%) | 16 (5%) |
| Worker^b^ | 30 (4%) | 26 (5%) | 21 (7%) |
| **Approximate number of full- and part-time employees during recruitment period**^c^ | ~500 | ~300 | ~250 |

^a^Professional refers to an employee involved with the design, development, and implementation of policies, programs, guidelines, systems, agreements, laws or regulations. (e.g., healthcare providers, probation agent, teacher, community worker, researcher, information technology, and pastoral).

^b^Worker refers to an employee performing manual work (e.g., kitchen, janitorial, building maintenance, and laundry).

^c^As per respective prison directors (personal communication between July and September 2021).

**Supplementary Table S2. Participants’ sociodemographic, clinical, and carceral characteristics stratified by prison occupation.**

|  | **Administration (n=59)** | **CO**  **(n=317)** | **HCP**  **(n=39)** | **Kitchen**  **(n=32)** | **Manager (n=80)** | **Other^h^**  **(n=70)** | **Overall (N=600)** |
| --- | --- | --- | --- | --- | --- | --- | --- |
| **Sociodemographic characteristics** |  |  |  |  |  |  |  |
| **Age, years** |  |  |  |  |  |  |  |
| Median (Q1-Q3) | 43 (35-53) | 43 (33-50) | 38 (32-48) | 51 (41-57) | 46 (38-51) | 42 (30-50) | 43 (33-51) |
| **Age category – n (%)** |  |  |  |  |  |  |  |
| 18-29 | 11 (18.6%) | 46 (14.5%) | 7 (17.9%) | 4 (12.5%) | 2 (2.5%) | 16 (22.9%) | 86 (14.3%) |
| 30-39 | 11 (18.6%) | 81 (25.6%) | 13 (33.3%) | 3 (9.4%) | 20 (25.0%) | 14 (20.0%) | 142 (23.7%) |
| 40-49 | 16 (27.1%) | 101 (31.9%) | 10 (25.6%) | 8 (25.0%) | 32 (40.0%) | 21 (30.0%) | 189 (31.5%) |
| 50 and over | 21 (35.6%) | 88 (27.8%) | 9 (23.1%) | 17 (53.1%) | 26 (32.5%) | 18 (25.7%) | 181 (30.2%) |
| *Missing* | 0 (0%) | 1 (0.3%) | 0 (0%) | 0 (0%) | 0 (0%) | 1 (1.4%) | 2 (0.3%) |
| **Sex – n (%)** |  |  |  |  |  |  |  |
| Male | 7 (11.9%) | 160 (50.5%) | 12 (30.8%) | 21 (65.6%) | 42 (52.5%) | 27 (38.6%) | 271 (45.2%) |
| Female | 52 (88.1%) | 155 (48.9%) | 27 (69.2%) | 10 (31.3%) | 38 (47.5%) | 43 (61.4%) | 326 (54.3%) |
| *Missing* | 0 (0%) | 2 (0.6%) | 0 (0%) | 1 (3.1%) | 0 (0%) | 0 (0%) | 3 (0.5%) |
| **Race/ethnicity – n (%)** |  |  |  |  |  |  |  |
| White | 44 (74.6%) | 235 (74.1%) | 28 (71.8%) | 22 (68.8%) | 71 (88.8%) | 49 (70.0%) | 451 (75.2%) |
| Visible minority^a^ | 15 (25.4%) | 74 (23.3%) | 11 (28.2%) | 8 (25.0%) | 9 (11.3%) | 20 (28.6%) | 137 (22.8%) |
| *Missing* | 0 (0%) | 8 (2.5%) | 0 (0%) | 2 (6.3%) | 0 (0%) | 1 (1.4%) | 12 (2.0%) |
| **Education level – n (%)** |  |  |  |  |  |  |  |
| High school or trade certificate | 21 (35.6%) | 43 (13.6%) | 11 (28.2%) | 23 (71.9%) | 10 (12.5%) | 23 (32.9%) | 132 (22.0%) |
| College diploma | 25 (42.4%) | 167 (52.7%) | 14 (35.9%) | 5 (15.6%) | 37 (46.3%) | 7 (10.0%) | 256 (42.7%) |
| University or higher | 12 (20.3%) | 101 (31.9%) | 14 (35.9%) | 2 (6.3%) | 33 (41.3%) | 38 (54.3%) | 200 (33.3%) |
| *Missing* | 1 (1.7%) | 6 (1.9%) | 0 (0%) | 2 (6.3%) | 0 (0%) | 2 (2.9%) | 12 (2.0%) |
| **Personal gross yearly income^b^ (CAD) – n (%)** |  |  |  |  |  |  |  |
| Less than $60,000 | 49 (83.1%) | 23 (7.3%) | 6 (15.4%) | 24 (75.0%) | 2 (2.5%) | 39 (55.7%) | 144 (24.0%) |
| $60,000-$89,999 | 4 (6.8%) | 215 (67.8%) | 21 (53.8%) | 4 (12.5%) | 33 (41.3%) | 19 (27.1%) | 297 (49.5%) |
| $90,000 or more | 0 (0%) | 63 (19.9%) | 12 (30.8%) | 0 (0%) | 44 (55.0%) | 4 (5.7%) | 123 (20.5%) |
| *Missing* | 6 (10.2%) | 16 (5.0%) | 0 (0%) | 4 (12.5%) | 1 (1.3%) | 8 (11.4%) | 36 (6.0%) |
| **Clinical characteristics** |  |  |  |  |  |  |  |
| **SARS-CoV-2 serology – n (%)** |  |  |  |  |  |  |  |
| Negative | 52 (88.1%) | 247 (77.9%) | 31 (79.5%) | 28 (87.5%) | 68 (85.0%) | 66 (94.3%) | 495 (82.5%) |
| Positive | 7 (11.9%) | 70 (22.1%) | 8 (20.5%) | 4 (12.5%) | 12 (15.0%) | 4 (5.7%) | 105 (17.5%) |
| **History of PCR testing – n (%)** |  |  |  |  |  |  |  |
| No PCR test | 11 (18.6%) | 36 (11%) | 3 (8%) | 8 (25%) | 8 (10%) | 8 (12%) | 75 (12.5%) |
| Negative PCR test | 46 (78.0%) | 221 (70%) | 28 (72%) | 21 (66%) | 64 (80%) | 58 (85%) | 441 (73.5%) |
| Positive PCR test | 2 (3.4%) | 58 (18%) | 8 (20%) | 3 (9%) | 8 (10%) | 2 (3%) | 81 (13.5%) |
| *Missing* | 0 (0%) | 2 (1%) | 0 (0%) | 0 (0%) | 0 (0%) | 0 (0%) | 3 (0.5%) |
| **History of COVID-19 symptoms^c^ – n (%)** |  |  |  |  |  |  |  |
| No | 15 (25.4%) | 96 (30.3%) | 8 (20.5%) | 16 (50.0%) | 31 (38.8%) | 28 (40.0%) | 194 (32.3%) |
| Yes | 43 (72.9%) | 220 (69.4%) | 30 (76.9%) | 16 (50.0%) | 48 (60.0%) | 41 (58.6%) | 400 (66.7%) |
| *Missing* | 1 (1.7%) | 1 (0.3%) | 1 (2.6%) | 0 (0%) | 1 (1.3%) | 1 (1.4%) | 6 (1.0%) |
| **Medical comorbidities^d^ – n (%)** |  |  |  |  |  |  |  |
| None | 27 (46%) | 164 (52%) | 19 (49%) | 17 (53%) | 39 (49%) | 28 (40%) | 294 (49%) |
| 1 | 19 (32%) | 85 (27%) | 13 (33%) | 7 (22%) | 27 (34%) | 28 (40%) | 179 (30 %) |
| 2 or more | 13 (22%) | 60 (19%) | 6 (15%) | 6 (19%) | 14 (17%) | 12 (17%) | 111 (18%) |
| *Missing* | 0 (0%) | 8 (2%) | 1 (3%) | 2 (6%) | 0 (0%) | 2 (3%) | 16 (3%) |
| **COVID-19 vaccination^e^ – n (%)** |  |  |  |  |  |  |  |
| Not vaccinated | 4 (7%) | 58 (18%) | 4 (10%) | 2 (6%) | 2 (3%) | 4 (6%) | 74 (12%) |
| 1 dose | 6 (10%) | 52 (16%) | 3 (8%) | 5 (16%) | 5 (6%) | 10 (14%) | 82 (14%) |
| 2 doses | 47 (80%) | 199 (63%) | 32 (82%) | 23 (72%) | 73 (91%) | 56 (80%) | 430 (72%) |
| *Missing* | 2 (3%) | 8 (3%) | 0 (0%) | 2 (6%) | 0 (0%) | 0 (0%) | 14 (2%) |
| **COVID-19 vaccine status** **– n (%)** |  |  |  |  |  |  |  |
| Not fully vaccinated | 9 (15%) | 91 (29%) | 4 (10%) | 6 (19%) | 5 (6%) | 14 (20%) | 130 (22%) |
| Fully vaccinated | 48 (82%) | 218 (69%) | 35 (90%) | 24 (75%) | 75 (94%) | 56 (80%) | 456 (76%) |
| *Missing* | *2 (3%)* | *8 (2%)* | *0 (0%)* | *2 (6%)* | *0 (0%)* | *0 (0%)* | *14 (2%)* |
| **Carceral characteristics** |  |  |  |  |  |  |  |
| **Prison – n (%)** |  |  |  |  |  |  |  |
| EDRDP | 18 (30.5%) | 79 (24.9%) | 16 (41.0%) | 9 (28.1%) | 22 (27.5%) | 17 (24.3%) | 163 (27.2%) |
| EDM | 28 (47.5%) | 181 (57.1%) | 12 (30.8%) | 16 (50.0%) | 36 (45.0%) | 36 (51.4%) | 310 (51.7%) |
| EDSJ | 13 (22.0%) | 57 (18.0%) | 11 (28.2%) | 7 (21.9%) | 22 (27.5%) | 17 (24.3%) | 127 (21.2%) |
| **Number of days of employment in prison per month – n (%)** |  |  |  |  |  |  |  |
| < 21 | 28 (47.5%) | 132 (41.6%) | 17 (43.6%) | 16 (50.0%) | 25 (31.3%) | 38 (54.3%) | 256 (42.7%) |
| 21-27 | 20 (33.9%) | 147 (46.4%) | 12 (30.8%) | 15 (46.9%) | 51 (63.8%) | 24 (34.3%) | 269 (44.8%) |
| ≥ 28 | 11 (18.6%) | 33 (10.4%) | 10 (25.6%) | 1 (3.1%) | 3 (3.8%) | 6 (8.6%) | 64 (10.7%) |
| *Missing* | 0 (0%) | 5 (1.6%) | 0 (0%) | 0 (0%) | 1 (1.3%) | 2 (2.9%) | 11 (1.8%) |
| **Meal consumption in prison^e^ – n (%)** |  |  |  |  |  |  |  |
| Alone | 24 (40.7%) | 80 (25.2%) | 12 (30.8%) | 17 (53.1%) | 37 (46.3%) | 24 (34.3%) | 196 (32.7%) |
| With others | 33 (55.9%) | 232 (73.2%) | 27 (69.2%) | 14 (43.8%) | 43 (53.8%) | 43 (61.4%) | 392 (65.3%) |
| *Missing* | 2 (3.4%) | 5 (1.6%) | 0 (0%) | 1 (3.1%) | 0 (0%) | 3 (4.3%) | 12 (2.0%) |
| **Direct daily contact with people in prison^f^ – n (%)** |  |  |  |  |  |  |  |
| <10% | 57 (96.6%) | 134 (42.3%) | 13 (33.3%) | 21 (65.6%) | 60 (75.0%) | 36 (51.4%) | 322 (53.7%) |
| 10-49% | 1 (1.7%) | 87 (27.4%) | 12 (30.8%) | 4 (12.5%) | 18 (22.5%) | 24 (34.3%) | 146 (24.3%) |
| >50% | 1 (1.7%) | 96 (30.3%) | 14 (35.9%) | 7 (21.9%) | 2 (2.5%) | 10 (14.3%) | 132 (22.0%) |
| **Ability to physically distance in prison^g^ – n (%)** |  |  |  |  |  |  |  |
| Always | 32 (54.2%) | 25 (7.9%) | 1 (2.6%) | 10 (31.3%) | 20 (25.0%) | 19 (27.1%) | 107 (17.8%) |
| Almost always | 22 (37.3%) | 99 (31.2%) | 17 (43.6%) | 12 (37.5%) | 36 (45.0%) | 36 (51.4%) | 222 (37.0%) |
| Sometimes | 3 (5.1%) | 78 (24.6%) | 12 (30.8%) | 4 (12.5%) | 15 (18.8%) | 9 (12.9%) | 122 (20.3%) |
| Rarely | 2 (3.4%) | 110 (34.7%) | 9 (23.1%) | 4 (12.5%) | 9 (11.3%) | 4 (5.7%) | 138 (23.0%) |
| *Missing* | 0 (0%) | 5 (1.6%) | 0 (0%) | 2 (6.3%) | 0 (0%) | 2 (2.9%) | 11 (1.8%) |
| **Perceived concern of SARS-CoV-2 acquisition from others in prison – n (%)** |  |  |  |  |  |  |  |
| Somewhat or extremely worried | 21 (35.6%) | 136 (42.9%) | 11 (28.2%) | 14 (43.8%) | 32 (40.0%) | 26 (37.1%) | 242 (40.3%) |
| Neutral | 12 (20.3%) | 64 (20.2%) | 10 (25.6%) | 7 (21.9%) | 15 (18.8%) | 18 (25.7%) | 127 (21.2%) |
| Not or hardly worried | 26 (44.1%) | 116 (36.6%) | 18 (46.2%) | 11 (34.4%) | 33 (41.3%) | 26 (37.1%) | 230 (38.3%) |
| *Missing* | 0 (0%) | 1 (0.3%) | 0 (0%) | 0 (0%) | 0 (0%) | 0 (0%) | 1 (0.2%) |
|  |  |  |  |  |  |  |  |

CO: Correctional officer; EDM: Établissement de détention de Montréal; EDRDP: Établissement de détention de Rivière-des-Prairies; EDSJ: Établissement de détention de Saint-Jérôme; HCP: healthcare providers.

^a^Includes Black, Latin American, Arab, Asian, Indigenous, and Asian

^b^Refers to total annual income (CAD) from all paid work and all other sources before taxes and other deductions in the year prior to incarceration.

^c^Includes fever, chills, headache, sore throat, new or worsening cough, stuffy nose/congestion, difficulty breathing/shortness of breath, loss of smell or taste, fatigue, weakness, confusion, diarrhea, muscle pain, vomiting and nausea.

^d^Includes hypertension, diabetes, obesity (based on body mass index), asthma, chronic lung disease, chronic heart disease, chronic kidney disease, liver disease, cancer, chronic blood disorder, chronic neurological disorder, immunocompromised (HIV), immunocompromised (Other). ^e^Defined as the presence/absence of meal sharing with other correctional employees from same or different sectors while working in prison.

^f^Defined as being within less than two meters for at least 10 minutes, with or without a mask.

^g^Defined as whether the prison environment allows for physical distancing with other prison staff and incarcerated individuals (i.e., enough space to keep safe distance from other people, plexi-glass barriers).

^h^Includes probation agents, community workers, janitorial staff, building maintenance, teachers, pastoral, laundry, library, researchers, information technology, and students.

**
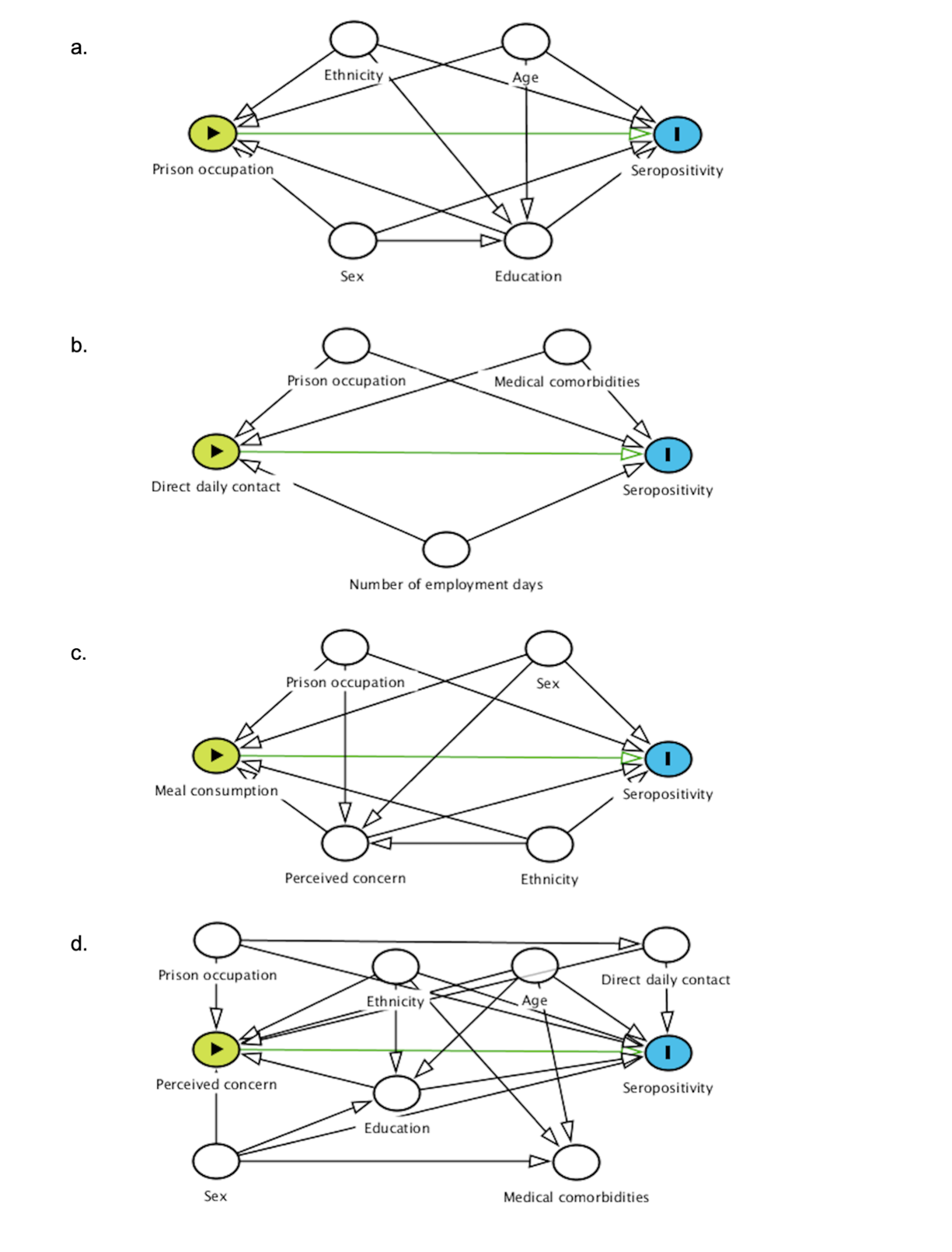
**

**Supplementary Figure S1: Direct acyclic graphs of the relationships between the carceral exposure of interest on SARS-CoV-2 seropositivity.**

Directed acyclic graphs (DAGs) depicting known or plausible relationship between the carceral exposure of interest on SARS-CoV-2 seropositivity. a. Prison occupation, adjusting for age, sex, ethnicity and education (Model 1); b. Meal consumption in prison, adjusting for sex, ethnicity, prison occupation and perceived concern of SARS-CoV-2 acquisition from others (Model 2); c. Direct daily contact with people in prison, adjusting for medical comorbidities, prison occupation, and number of days of employment in prison per month (Model 3); d. Perceived concern of SARS-CoV-2 acquisition from others in prison, adjusted for age, sex, ethnicity, education level, medical comorbidities, prison occupation, and direct daily contact with people in prison (Model 4).

**References**

1. Ministère de la Sécurité publique du Québec. Étude des crédits 2019–2020 (2019). <https://www.securitepublique.gouv.qc.ca/fileadmin/Documents/ministere/diffusion/etude_cr%C3%A9dits_TomeI_tomeII_2019-2020.pdf> [Accessed 30 May 2022].
2. Gouvernement du Québec. Situation COVID-19 – Établissement de détention (2022). <https://www.quebec.ca/sante/problemes-de-sante/a-z/coronavirus-2019/situation-coronavirus-quebec/situation-covid-19-etablissements-de-detention> [Accessed 30 May 2022].
